# Relation of Food Insecurity and Hemoglobin Level in Preschool Aged Children

**DOI:** 10.1155/2018/3950687

**Published:** 2018-01-15

**Authors:** Élida Mara Braga Rocha, Luiz Carlos de Abreu, Amanda Forster Lopes, Claudio Leone, Patrícia Dore Vieira, Italla Maria Pinheiro Bezerra, Sophia Cornbluth Szarfarc

**Affiliations:** ^1^Department of Nutrition, School of Public Health, University of São Paulo, São Paulo, SP, Brazil; ^2^Design of Studies and Scientific Writing Laboratory, The ABC School of Medicine, Santo André, SP, Brazil; ^3^Department of Maternal and Child Health, School of Public Health, University of São Paulo, São Paulo, SP, Brazil; ^4^Postgraduate Program in Public Policies and Local Development, School of Sciences of Santa Casa de Misericordia de Vitoria, Vitória, ES, Brazil

## Abstract

**Background:**

The iron deficiency anemia is a worldwide public problem, especially in developing countries, related to increased body needs and inadequate supply of iron from the diet.

**Objective:**

To analyze the association of food insecurity with hemoglobin concentration and the prevalence of anemia in preschool aged children in the city of Taubaté, São Paulo, Brazil.

**Methods:**

A cross-sectional study was conducted with 306 children of preschool age. The nutritional status was assessed according to hemoglobin level and anthropometric indicators. Socioeconomic data and Brazilian Household Food Insecurity Measurement Scale (EBIA) results were obtained from interview with parents.

**Results:**

The prevalence of anemia was around 19% of preschool aged children and 41.2% families presented food insecurity. The anthropometric indicators were not associated with food insecurity and even though the bivariate analysis demonstrated that mild food insecurity affects the hemoglobin level, after adjusting the multivariate model this association lost significance (*p* > 0.05).

**Conclusion:**

The prevalence of anemia of 19.2% and the household food insecurity was found among 42.2% of the population.

## 1. Background

Demographic, economic, and social alterations have triggered a new epidemiological configuration, partly because of changes in the nutritional profile [1] and lifestyle of the population, which also result in changes in the known associations between anthropometric indices and nutritional deficiencies.

This dynamism of nutritional inequalities that reflects the level of development of a society requires the addition of new instruments to evaluate the health status of the population. Therefore, some studies have suggested that the evaluation of food insecurity can be the best indicator of social and nutritional inequalities, mainly through enabling identification of socially vulnerable groups, among others [2–4], since that food security involves the health and well-being of the individual in their biological, economic, social, environmental, and cultural aspects [5].

Thus, in Brazil, in addition to ensuring the human right to adequate food, food security is the concept of a healthy, affordable, quality diet in sufficient quantity, in a permanent manner, without compromising access to other essential needs, based on healthy eating habits, respecting cultural diversity, being sustainable from a socioeconomic and agroecological point of view [6].

Although the Brazilian surveys in recent years indicate improvements in living and health conditions [7, 8], children under five years of age still present high prevalence of food insecurity [9].

It is clear that the most serious manifestations of food insecurity are hunger and malnutrition; however within these are “several hungers”: such as acute hunger or food urgency and chronic hunger or daily energy failure, which are also equivalent to the malnutrition or undernourishment named “hidden hunger,” characterized by quantitative and qualitative inadequacy of food [10], causing serious nutritional deficiencies.

The theoretical model that directs the researches in this subject is based on the premise that economic restriction causes food insecurity, limited access to food in adequate quality or sufficient quantity, and, consequently, nutritional disorders, such as macro- and micronutrient deficiency [11–22].

Among these dietary deficiencies, the most commonly investigated one is iron deficiency anemia, affecting around 800 million children and women, making it a worldwide public health problem, especially in developing countries. In children of preschool age from six months to five years old, the situation is more alarming, since the prevalence of anemia is 42.6%, affecting 273.2 million children worldwide [23].

Moreover, it cannot be overlooked that the consequences of anemia for the country harm not only children but also the entire population. The high indirect cost of the prematurity, the low birth weight and the prejudice to the cognitive development that occurs in infancy and is reflected in the course of life, have a substantial burden on the country's economy. Especially in developing countries, the monetary losses provided by the mathematical models add to the social losses due to anemia [24–26].

In this context, studying the relationship between anemia with situation of food insecurity is important. However few investigations in Brazil have considered these variables together [16–18], leaving a gap in the knowledge on the subject avoiding the construction of efficient intervention strategies.

Thus, the purpose of this article is to analyze the association of food insecurity with hemoglobin level and the prevalence of anemia in preschool age children.

## 2. Materials and Methods

### 2.1. Study Design

This is a cross-sectional study of preschool children between 24 and 48 months of age, enrolled in public daycare centers in the city of Taubaté, São Paulo, in 2014.

The city of Taubaté was chosen due to the importance of knowing the situation of food insecurity and its relation to nutritional deficiencies in children of preschool age who are residents of a locality with good indicators of health, education, and income and thus with the prospect of ideal conditions expected for growth and development, allowing identification of a different situation with respect to populations of disadvantaged areas.

Sampling was carried out through the Occupation, Income and Education Research, for which the city of Taubaté was divided into five regions [27] and subsequently grouped into two distinct socioeconomic groups: a vulnerable region with an average family income of up to 1.35 times the minimum wage and a wealthy region with income of more than 1.35 times the minimum wage per month. The sample size, of 290 children, was calculated on the assumption that differences in hemoglobin (Hb) level between children in the vulnerable and wealthy regions would be equivalent to 1/3 of the standard deviation of the average Hb of the healthy population, adopting *α* = 5% and *β* = 20%.

### 2.2. Data Collection Instruments

Socioeconomic information was obtained through a questionnaire sent to those responsible for the children and consisted of the following information: age and sex of the child, daycare center time, maternal education in complete years of schooling, number of inhabitants in the household, family income, and receipt of government social assistance (cash transfer program).

The food insecurity (FI) was assessed through the Brazilian Household Food Insecurity Measurement Scale (EBIA) [28]; based on the total number of affirmative answers for EBIA the households were classified into the following [9, 29]: food security (0 points), mild food insecurity (1 to 5 points), moderate food insecurity (6 to 9 points), and severe food insecurity (10 to 14 points).

Anthropometric measurements were performed in accordance with the SISVAN Anthropometric Indicators Measurement Guide [30] for weight and height measurements. Each child's weight-for-height, height-for-age, and weight-for-age* Z*-scores were calculated using the WHO Growth Standards. Wasting, stunting, and underweight were defined according to a cutoff of 0–2 of the respective* Z*-score. Overweight was defined as a* Z*-score greater than +2, according to the WHO curves [31].

The Hb level of the child was evaluated using a portable hemoglobinometer, Agabe® (Exa-M, Mogi da Cruzes, SP), and anemia was identified by the value of Hb <11.0 g/dL [23].

### 2.3. Statistical Analysis

All analyses were performed with the aid of the Statistical Package for Social Sciences (SPSS, Chicago, IL, USA). Descriptive analysis was realized to summarize the household food insecurity, hemoglobin level, and anthropometric indicators of child growth. The association between food insecurity and each variable was analyzed using binary analysis. First, the comparison of proportions of socioeconomic and biological variables with the food security and insecurity status was performed using the chi-squared test. Second, regarding the analysis of association between food insecurity and child nutritional status, related to anthropometric variables and Hb level, the Student *t*-test was used and the medians were compared with the Mann–Whitney test.

The variables that presented association with food insecurity at a level of significance less than or equal to 20%  (*p* < 0.20) were selected to compose the multivariate regression analysis. For the multiple model, the Odds Ratio (OR) and respective 95% confidence intervals (95% CI) were calculated, after adjusting the following variables: maternal education, income per capita, cash transfer program, and sex of the child. The adjusted OR and 95% CI from mild FI and moderate/severe FI were shown in table. Thus, the final model contained only the strongest associated variables with statistical significance of *p* < 0.05.

### 2.4. Ethical Considerations

Informed consent was obtained from all the children's guardians. The study was approved by the Ethics Committee of the School of Public Health, University of São Paulo (number 773287).

## 3. Results

The nutritional profile of the children and the situation of food insecurity in their families are presented in Table 1. Regarding anthropometric indicators, among the nutritional disorders, the prevalence of overweight (6.2%) for the indicator weight/age when compared to the height/age deficit (3.2%) stands out, since anemia was present in 19.2% of preschool children.

In relation to the families studied, 41.2% were classified in the food insecurity category, the most prevalent in a mild FI form (26.6%), followed by moderate FI (12.7%), and finally severe FI (1.9%); the food security condition (58.8%) therefore predominated.  Table 2 allows verification of the low maternal education, per capita income and receipt of government social assistance among households that presented food insecurity (*p* < 0.001).

**Figure 1:**
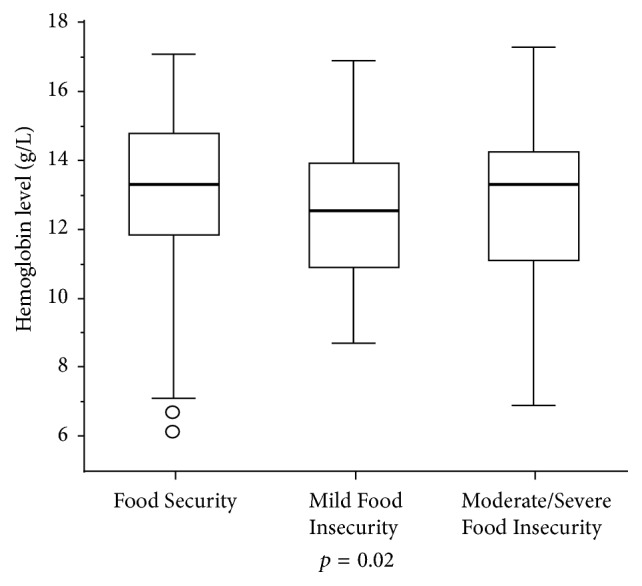
Hemoglobin level and household food insecurity status. Taubaté, São Paulo, Brazil, 2014.

Regarding the nutritional consequences, association was not found of FI with growth indicators which consider age, height, and weight; *p* > 0.05 (Table 2), but the Hb level was lower among children from families in mild FI; *p* < 0.05 (Figure 1).

Thus, children in mild FI have 1.9 times more risk of presenting anemia compared to those with food security (95% CI: 1.01 to 3.6). However, the multivariate regression (Table 3) did not demonstrate the same association after adjustment of the model (*p* > 0.05).

## 4. Discussion

The studies investigating the relationship between food insecurity and the Hb level in children under five years of age in low [11–15], medium [16–18], and high income families [19–22] have found distinct and complex results. The conflicting evidence can be grouped into two conclusion blocks: those who found no significant association between household food insecurity and alterations in Hb levels in children [12–20] and, on the other hand, those who demonstrated an increased risk of childhood anemia among families with food insecurity [11, 21, 22].

Our results showed that food insecurity increased the likelihood of children of preschool age presenting anemia; however after adjusting for variables this association lost power of significance, comparable to the results of studies that investigated aboriginal communities in Canada with a high prevalence of severe food insecurity [19, 20]. Similarly, in locations with a high prevalence of childhood anemia and severe food insecurity where it was expected to find an association between these variables, they were not described, for example, in Asia [12, 13] and Africa [14, 15].

Studies in the northeast of Brazil have found no association between food insecurity and nutritional deficiency [16, 17], which confirm the findings of this research in the southeast region of the country, demonstrating that even in different socioeconomic situations in the same country it was not possible to make relationship of food insecurity with biochemical levels or anthropometric indicators.

Paradoxically, in the United States, where levels of food insecurity are low, studies have reported that low-income children with food insecurity have twice the risk of presenting anemia compared to those with food security [21, 22], this being similar to data analyzed in India, a country that faces major socioeconomic inequalities [11].

This disparity in situations requires an analysis of the concept of the study variables and thus it is important to stress that iron deficiency anemia has a “pansocial” character that affects rich and poor countries; the main determinant of this nutritional deficiency is low intake of foods rich in iron and/or high physiological need, causing a negative balance in food consumption of the mineral and subsequently anemia [23]. In another context, food insecurity is strongly linked to household financial conditions, this being the primary indicator of access to adequate and sufficient food. Thus, the relationship between food insecurity and the wealth index has an important association with infant anemia [11, 21].

Global and regional situations presented in the scientific literature seem to point to distinct mechanisms of defense against nutritional deficiencies among families with food insecurity, the result of a complex interaction that can be perceived in some locations and not others. McDonald et al. [12] investigating mothers and children in rural Cambodia found an association between anemia and maternal malnutrition with increasing severity of food insecurity, but not among the children, suggesting that the mother restricted both the quality and the quantity of food she consumed in favor of voluntarily offering it to the younger individuals in the family. According to the same, authors [12], this conception of unequal distribution of intrafamilial food, prioritizing a healthy and balanced diet for some rather than others, is based on the cultural characteristic of women having the main responsibility for the management of food resources within the family, leading to a significant and positive impact on future generations, such as the smaller children [32, 33].

In this context, researches show a positive relationship between the family affective bonding and child nutritional status [34], even in low-income situations, indicating that the mother-child bonding, when adequate, can have a protective effect against child malnutrition [35]. So, it can be assumed that the lack of association between food insecurity and childhood anemia in some locations may be the result of proper functioning of the family mechanisms that preserve a traditional diet or indicative of a still ongoing nutritional transition regarding the modification of food consumption patterns.

Among those researches in which anemia with increased severe food insecurity is demonstrated, this may be due to extreme poverty in that the family protection fails or a situation where the diet of ultraprocessed food predominates, causing low micronutrient intake. Some studies [15, 20] point out evidence that households with food insecurity that do not maintain adequate diet in animal protein among children increase the risk of childhood anemia. Confirming this idea, the results for an area with a high prevalence of food insecurity in Rio de Janeiro showed that children under three years of age with moderate and severe food insecurity presented inadequate protein intake and an iron deficiency [36].

This divergence of findings does not detract from the EBIA perception scale, and it only demonstrates that the associations of socioeconomic, health, and nutrition variables are complex and should be continuously investigated and discussed with other scientific evidence. However, it should be clarified that EBIA is a valid and essential indicator for public policies in Brazil, mainly to track and monitor the vulnerability of beneficiaries of social programs [37].

Regarding the limitations of the study, it should be mentioned that the cross-sectional study has a difficulty regarding the temporal relationship between the variables, and even if it is possible to perform a test of association between the risk factor and the outcome in question, we can not prove the causal relationship, since the exposure data and the outcome were collected simultaneously in a single time cut. Another issue is when investigating low prevalence conditions, which would imply the need for a relatively large sample. But this effect can be reversed when the sampling process was well delimited and representative of the population, as is the case of the Taubaté research that divided the total number of daycare centers into two regions (vulnerable and wealthy economically), according to family income, making the randomization of institutions in a well-defined and rigorous way possible, therefore with a sample of preschool children significantly represented. We emphasize that the cross-sectional study is a useful tool for the identification of risk groups and for health action and planning.

It is worth noting that anemia and nutrient deficiencies slow down the process of child growth and development [24, 25]. Therefore, this reality can be countered by the effective application of public and social policies aimed at providing safe and adequate nutrition [38–40].

No statistically significant association was found between food insecurity and the Hb level and anthropometric indicators do not disqualify the Brazilian Household Food Insecurity Measurement Scale but point to its principal characteristic, a method of measuring the individual's perception of the issue of access to food, passing from the fear to the fact of experiencing real hunger. Thus, the analysis should be viewed with caution, as well as being described and discussed in a critical way for consideration of the results of food insecurity, which in conjunction with other indicators to evaluate health is an important initiative to determine the level of quality of life of the population and enable indirect mapping of the level of social and economic development of a society.

## 5. Conclusion

The prevalence of anemia of 19.2% and the household food insecurity was found among 42.2% of the population.

## Figures and Tables

**Table 1:** Nutritional status of preschool children, according to anthropometric indicators, hemoglobin level, and household food insecurity. Taubaté, São Paulo, Brazil, 2014.

Variables	*n*	%
*Household food insecurity*		
Severe food insecurity	6	1,9
Moderate food insecurity	39	12,7
Mild food insecurity	82	26,6
Food security	181	58,8
*Hemoglobin level*		
<11 g/dL	59	19,2
≥11 g/dL	249	80,8
*Child growth*		
Wasting^*∗*^	3	1,0
Stunting^†^	10	3,2
Underweight^‡^	5	1,6
Overweight^§^	19	6,2

^*∗*^Weight-for-length *Z*-scores less than −2,00. ^†^Length-for-age *Z*-scores less than −2,00. ^‡^Weight-for-age *Z*-scores less than −2,00. ^§^Weight-for-age *Z*-scores greater than +2,00.

**Table 2:** Socioeconomic and demographic characteristics association with household food insecurity status. Taubaté, São Paulo, Brazil, 2014.

Variables	Food security	Food insecurity	*p* values
	*n* (%)	*n* (%)	
*Socioeconomic characteristics*			
Maternal education			
≤8 years	41 (22,7)	59 (46,8)	<0,001
>8 years	140 (77,3)	67 (53,2)	
Income per capita			
<0,25 wage	19 (10,7)	36 (29)	<0,001
≥0,25 wage	158 (89,3)	88 (71,0)	
Cash transfer program			
Yes	36 (19,9)	54 (42,5)	<0,001
No	145 (80,1)	73 (57,5)	
*Child characteristics*			
Child mean age in months	37,6 ± 6,9 SD	38,7 ± 7,0 SD	0,913
Sex			
Male	71 (39,2)	69 (54,3)	0,009
Female	110 (60,8)	58 (45,7)	
Permanence daycare center			
Partial time (4 hours)	85 (47)	66 (52)	0,387
Full time (8 hours)	96 (53)	61 (48)	
*Nutritional status of children*			
Hemoglobin level (g/dL)	13,2 ± 2,1 SD	12,6 ± 2,0 SD	0,01
Weight-for-length (*Z*-scores)	0,43 ± 1,0 SD	0,56 ± 1,1 SD	0,296
Length-for-age (*Z*-scores)	0,05 ± 1,0 SD	−0,16 ± 1,0 SD	0,076
Weight-for-age (*Z*-scores)	0,33 ± 1,0 SD	0,31 ± 1,1 SD	0,816

Chi-Square Test, Student *t-*test, Mann–Whitney test, wage: US$ 306; and SD: standard deviation.

**Table 3:** Multivariate regression analysis of socioeconomics variables, child characteristics, and hemoglobin level and household food insecurity status. Taubaté, São Paulo, Brazil, 2014.

Variables	Crude odds ratio (95% CI)				Adjusted odds ratio (95% CI)			
	Mild FI	*p*	Moderate/severe FI	*p*	Mild FI	*p*	Moderate/severe FI	*p*
*Maternal education*								
≤8 years	2.4 (1.3–4.1)	0.003	4.7 (2.4–9.3)	<0.001	2.0 (1.1–3.6)	0.03	2.6 (1.2–5.6)	0.01
>8 years	1		1		1		1	
*Income per capita*								
<0.25 wage	1.9 (0.9–4.0)	0.08	7.6 (3.5–16.2)	0.57			3.9 (1.7–9.1)	0.001
≥0.25 wage	1		1				1	
*Cash transfer program*								
Yes	2.2 (1.2–3.9)	0.01	5.0 (2.5–10.0)	<0.001	1.9 (1.0–3.5)	0.05	2.7 (1.2–5.8)	0.01
No	1		1		1		1	
*Sex*								
Male	1.5 (0.9–2.6)	0.10	2.5 (1.3–5.0)	0.01				
Female	1		1					
*Hemoglobin level*								
<11 g/dL	1.9 (1.0–3.6)	0.05	1.6 (0.7–3.5)	0.28				
≥11 g/dL	1		1					

Wage = US$ 306; FI: food insecurity; CI: confidence interval.

## Abbreviations Used

Hb: Hemoglobin
FI: Food insecurity
WHO: World Health Organization
EBIA: Brazilian Household Food Insecurity Measurement Scale.
